# High-throughput screening of filamentous fungi using nanoliter-range droplet-based microfluidics

**DOI:** 10.1038/srep27223

**Published:** 2016-06-07

**Authors:** Thomas Beneyton, I. Putu Mahendra Wijaya, Prexilia Postros, Majdi Najah, Pascal Leblond, Angélique Couvent, Estelle Mayot, Andrew D. Griffiths, Antoine Drevelle

**Affiliations:** 1 École Supérieure de Physique et de Chimie Industrielles de la Ville de Paris (ESPCI Paris), CNRS UMR 8231, 10, rue Vauquelin, 75231 Paris Cedex 05, France; 2 Ets J. Soufflet/CRIS-OSIRIS, Quai Sarrail, BP12, 10400 Nogent-sur-Seine, France

## Abstract

Filamentous fungi are an extremely important source of industrial enzymes because of their capacity to secrete large quantities of proteins. Currently, functional screening of fungi is associated with low throughput and high costs, which severely limits the discovery of novel enzymatic activities and better production strains. Here, we describe a nanoliter-range droplet-based microfluidic system specially adapted for the high-throughput sceening (HTS) of large filamentous fungi libraries for secreted enzyme activities. The platform allowed (i) compartmentalization of single spores in ~10 nl droplets, (ii) germination and mycelium growth and (iii) high-throughput sorting of fungi based on enzymatic activity. A 10^4^ clone UV-mutated library of *Aspergillus niger* was screened based on *α*-amylase activity in just 90 minutes. Active clones were enriched 196-fold after a single round of microfluidic HTS. The platform is a powerful tool for the development of new production strains with low cost, space and time footprint and should bring enormous benefit for improving the viability of biotechnological processes.

Filamentous fungi are arguably the preferred source of industrial enzymes because of their excellent capacity for extracellular protein production^1^: filamentous fungi naturally secrete large amounts of hydrolytic enzymes, including amylases, cellulases or proteases, that are involved in the biochemical degradation of biomass. In addition, due to their extraordinary metabolic versatility, filamentous fungi are also used for the production of a variety of other products such as organic acids and pharmaceuticals^2^,^3^. Improving production strains is crucial for the economic viability of industrial biotechnology. Whilst genetic engineering and omics tools have made substantial contributions to rational strain improvement^4^, the lack of rapid, generic systems for screening of filamentous fungi still severely limits the discovery of better production strains and new enzymatic activites. Currently, the most widely used and flexible screening methods are based on compartmentalizing clonal populations of filamentous fungi in microtiter plate wells. However, even using expensive automated colony pickers and liquid handling robots, throughput is limited to typically only ~100 fungi.h^−1^ because of difficulties in manipulating filamentous fungi^5^. Fluorescence-activated cell sorting (FACS) can analyze and sort cells at rates of up to 7 × 10^4^ cells.s^−1^ 6, but can only be used to sort filamentous fungi in the earlier stages of germination, owing to size incompatibility between filamentous fungi and the nozzle^7^. Futhermore, fluorescence is detected in a continuous aqueous stream^8^ and the absence of compartmentalization makes it impossible to screen based on the activity of secreted proteins or extracellular metabolites.

Recently, droplet-based microfluidics technology has allowed major advances for the screening of microorganisms by significantly increasing the throughput and enlarging the range of systems that can be selected. Highly monodisperse droplets of picolitre volume can be made, fused, injected, split, incubated and sorted triggered on fluorescence, at kHz frequencies^9^,^10^,^11^. Typically, single bacterial or yeast cells are compartmentalized in droplets of ~10 pl volume, allowing screening of enzymes expressed intracellularly^12^,^13^,^14^, on the surface of cells^15^ or secreted from cells^16^,^17^, with a 1,000-fold increase in speed and a 1-million-fold reduction in volume (and hence cost) compared to robotic microtiter plate-based systems^15^. Larger drops have also been used to grow yeast cells to screen for extracellular metabolite production or consumption (300 pL)^18^, or to screen hybridoma cells for monoclonal antibody production (660 pL)^19^, albeit at lower throughput (1–10 s^−1^). However, microfluidic HTS have never been used with filamentous fungi. Indeed, growing and screening filamentous fungi in droplets is challenging due to the apical growth of the hyphae after spore germination to form an expanding branched mycelia network^20^. As we show here, filamentous fungi cannot be screened in picoliter droplets in microfluidic systems: the hyphal tips, where most protein secretion occurs^21^, rapidly exit the droplets, leading to uncontrolled coalescence. This limits the incubation period to only a few hours, which is too short to allow secretion of enough enzyme for screening.

This work proposes a novel approach for the HTS of filamentous fungi using nanoliter-range droplet-based microfluidics tools. Single spores can be encapsulated in ~10 nl droplets (volume ~3 orders of magnitude larger than those typically used for sorting bacteria and yeast) which can be incubated and sorted based on fluorescence. This system allowed 24 h incubation of filamentous fungi (*Aspergillus niger*) and screening of ~7,000 fungi.h^−1^ based on the activity of a secreted enzyme (*α*-amylase). A whole-genome UV-mutated *A. niger* library was enriched 196-fold for fungi secreting *α*-amylase after one round of microfluidic HTS. Sorted clones were further analyzed using a robotic microplate based system at ~400 clone.h^−1^: 98.1% exhibited *α*-amylase activity. The HTS platform can easily be adapted to screen for other enzymatic activities.

## Results

### Nanoliter-range droplet-based microfluidic modules

**Figure 1:**
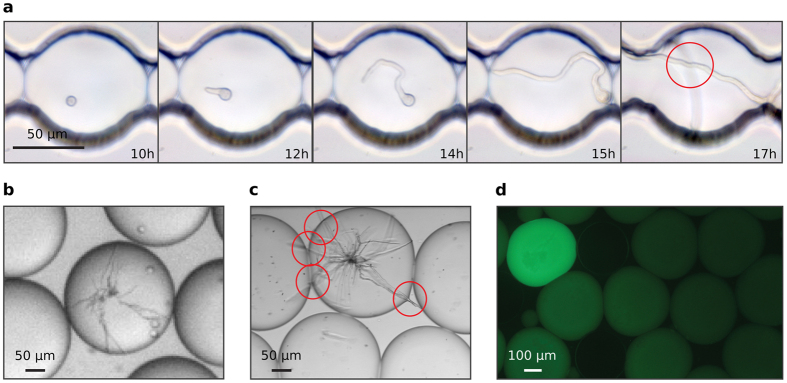
Growth of filamentous fungi in water in oil (w/o) droplets. (**a**) Microscopic image sequence (40×) over 17 h of a 250 pl droplet containing a single germinating spore. The droplet was immobilized in a drop spot chip^45^ and incubated at 30 °C. (**b**) Micrograph (15×) of 18 nl droplets after single spore encapsulation and 24 h incubation at 30 °C. (**c**) Micrograph (15×) of 18 nl droplets after single spore encapsulation and 32 h incubation at 30 °C. (**d**) Epifluorescence image (10×) of 18 nl droplets after single spore encapsulation with a fluorogenic substrate and 24 h incubation at 30 °C: the secreted *α*-amylase activity induces green fluorescence within droplets containing fungi. Red circles highlight the hyphal tips exiting the droplet.

Using nanoliter size droplets is essential for screening of filamentous fungi. Typically, in flasks, the time from which *Aspergillus* spores germinate to the stage where they start to display detectable enzymatic activity is ~24 h and optimum levels of activity are usually only reached after several days of incubation, depending on the secreted enzyme. The requirement for long incubation time combined with the rapid growth of the fungal hyphae makes screening filamentous fungi in picoliter droplets a challenging task: even in 250 pl droplets the hyphal tips exit the droplets in ~15 h, causing uncontrolled coalescence: (Fig. 1a, Supplementary Movie S1). In contrast, encapsulating single spores in 18 nl droplets allows growth of the branched mycelial network for up to 24 h confined in the droplet (Fig. 1b) with the hyphal tips exiting the droplets only after incubation for 32 h (Fig. 1c). Furthermore, after 24 h incubation, secreted *α*-amylase was easily detectable in the droplets using a fluorogenic substrate (Fig. 1d).

Increasing droplet volume from the pl to the nl range affects droplet formation, stability and dielectrophoretic sorting. It was straightforward to produce highly monodisperse (polydispersity ≤5% by volume) 10–20 nl droplets at 80–90 droplets.s^−1^ using a flow-focusing^22^ device with a constriction orifice 225 *μ*m wide and 250 *μ*m deep (Supplementary Fig. S1 and Movie S2), an oil phase of Novec HFE-7500 fluorinated oil containing 2.5% (w/w) KryJeffD_900_ (a triblock copolymer fluorosurfactant)^23^ and an aqueous phase of synthetic growth medium. Droplets were found to be highly stable and could be collected, incubated and reloaded. Analysis of the size distribution of 10 nl droplets after 24 h incubation at 30 °C in the form of a concentrated, creamed emulsion in the glass capillary and re-injection into the sorting chip (Supplementary Fig. S1) indicated that ≤1.0% of droplets were coalesced (Supplementary Fig. S2).

We developed a fluorescence-activated droplet sorting (FADS) device specifically adapted to sort nanoliter droplets. In dielectrophoretic droplet sorters^12^,^15^,^24^, droplets flow in carrier oil towards a Y-shaped junction. With no electric field, all drops flow into the waste channel which offers lower hydrodynamic resistance than the second, collect channel. To direct droplets into the collect channel, on chip electrodes are energized, creating an electrical field gradient, which generates a dielectrophoretic force (DEP) acting on the droplets. In order to be sorted, DEP forces must displace the droplet by a critical distance orthogonal to the flow, *d*_*o*_, in the time, *t*_*p*_, it takes the droplet to traverse the electrical field gradient. The DEP force on a spherical droplet is given by equation (1)^25^,


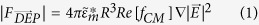


in which, |*F*_*DEP*_| is the magnitude of the DEP force, *R* is the radius of the droplet being subjected to the DEP force, *Re* [*f*_*CM*_] is the Claussius-Mosscoti factor (which itself depends on 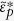 and 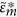: the complex permittivity of the droplet and the carrier fluid, respectively) and 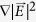 denotes the electrical field gradient. Assuming that d_0_ = 2*R*, and that, in order to prevent false positives, no more than one droplet can be in the electric field in the sorting chamber at any one time, the theoretical maximum sorting frequency, *f*_*sort*_, can be calculated (see Supplementary Information):


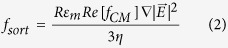


where *η* designates the viscosity of the carrier oil. Equation (2) shows that *f*_*sort*_ is directly proportional to *R* and to 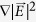, and that increasing *R* apparently favors DEP sorting as lower 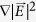 is required. However, larger droplets are more easily split by electric fields^26^. The electric field, *E*_max_, above which droplets split, when Maxwell stress surmounts the resistance to deformation due to interfacial tension, is given by the expression:


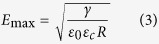


Therefore, the maximum sorting frequency is a function of both the magnitude, *E*, and inhomogeneity, 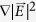, of the electric field in the sorter and depends on the volume of the drops and the size and geometry of the sorter.

**Figure 2:**
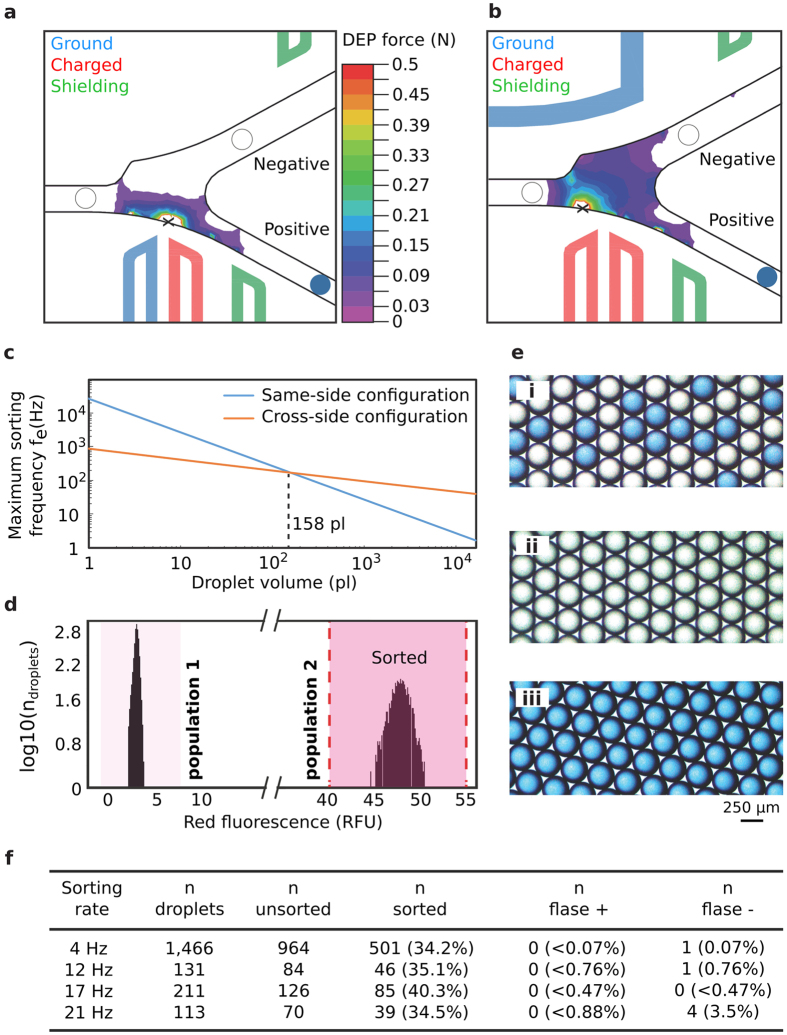
Validation of the sorting device. (**a**) Same-side electrode configuration: schematic and DEP force simulation (**b**) Cross-side electrode configuration: schematic and DEP force simulation. The corresponding electrical potential distributions for each electrode configuration are shown in Figure S2. (**c**) Calculated maximum sorting frequency *f*_*e*_ as a function of droplet volume for same-side and cross-side configurations. (**d**) Fluorescence histogram of a binary emulsion of 10 nl droplets containing 1 *μ*M sulforhodamine B (population 1, 65%) or 30 *μ*M sulforhodamine B plus blue ink (population 2, 35%) before sorting using the DEP sorter with the cross-side electrode configuration. The sorting gate was set to sort only droplets with red fluorescence between 40 and 55 RFU (vertical dashed lines) corresponding to population 2. (**e**) Color pictures of the emulsion before (i) and after sorting at 4 droplet.s^−1^ from the negative (ii) or positive (iii) channels. (**f**) Video analysis of the sorting efficiency at different throughputs.

We investigated two different electrode configurations: the first design had both charged and ground electrodes on the same side of the sorter (Fig. 2a), as used for fluorescence-activated sorting of pl droplets^12^,^15^ and the second design had the charged and ground electrodes on the opposite sides of the sorter (Fig. 2b). Finite element analysis (FEA) simulations indicated that in the same-side electrode configuration, although the DEP force was high between the positive and ground electrodes close to the channel wall adjacent to the electrodes, it drops off rapidly across the width of the channel (Fig. 2a and Supplementary Fig. S3). In contrast, using the cross-side electrode configuration the DEP force extends across the entire width of the sorting channel (Fig. 2b and Supplementary Fig. S3). By combining a model of the electric field distribution with equations (2) and (3) it was possible to calculate the maximum droplet sorting frequency limited by electrosplitting, *f*_*e*_, as a function of droplet volume (Fig. 2c; Supplementary Information). With both electrode configurations *f*_*e*_ is inversely proportional to the droplet volume. However, if the droplet volume is >158 pl (~67 *μ*m diameter), the cross-side electrode configuration allows the highest sorting rate, whereas with droplets of <158 pl the same-side electrode configuration allows the highest sorting rate.

The superiority of the cross-side electrode configuration for sorting 10 nl droplets was confirmed experimentally. Sorting of 10 nl droplets was achieved when AC electric field pulses of 1.4 kV_*pp*_, 30 kHz, 30 ms, were applied with the cross-side electrode configuration (Supplementary Movie S3). In contrast, the same electric field pulses could not sort droplets with the same-side electrode configuration (Supplementary Movie S4). The efficiency of the sorting device with the cross-side electrode configuration was further investigated by sorting 10 nl droplets of a binary emulsion comprising low red fluorescent droplets containing 1 *μ*M sulforhodamine B (negative; 65%) and highly red fluorescent droplets containing 30 *μ*M sulforhodamine B and blue ink (positive; 35%) (Fig. 2d). The false positive and false negative error rates were determined either by color imaging of the emulsion before and after sorting or by high speed video analysis of droplets trajectories during the sorting process at different throughputs. When droplets were sorted at 4 s^−1^, the emulsion collected in the positive channel was composed of 100% positive (blue) droplets while the emulsion collected in the negative channel contained 99.9% of negative droplets (Fig. 2e; Supplementary Fig. S3). Based on the video analysis of the sorting process (Supplementary Movie S5), the false positive and false negative error rates were found to less than 0.07% and equal to 0.07%, respectively (Fig. 2f). These low error rates validate the ability of the cross-side electrode configuration device to efficiently sort 10 nl droplets based on fluorescence. The few false negative errors were always due to instability in the reloading regime of the droplets, resulting in temporary modification of the spacing at the sorting junction. The device could operate at up to 21 droplets.s^−1^ with acceptable false positive and false negative error rates (Fig. 2f; Supplementary Movie S6), and was also able to sort 20 nl droplets with similar efficiency (Supplementary Fig. S3). This is close to the maximum theoretical sorting frequency, *f*_*e*_, for 10 nl droplets calculated from the model (Fig. 2c), which was 3 droplet.s^−1^ for same-side electrode configuration and 46 droplet.s^−1^ for the cross-side electrode configuration.

### Droplet-based microfluidics screening of filamentous fungi

#### 

**Figure 3:**
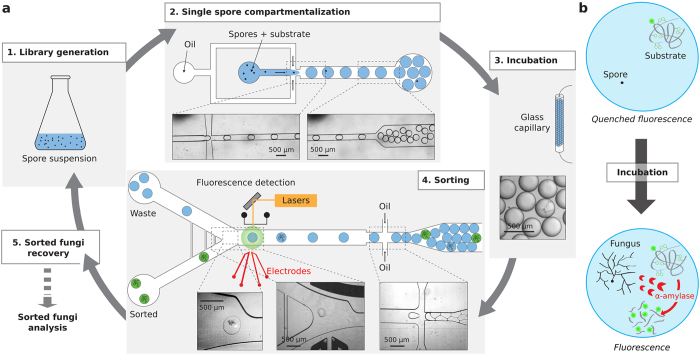
Droplet-based microfluidics screening platform. (**a**) Schematic of the system. 1. Generation of a whole-genome mutated fungi library. 2. The spores are compartmentalized in ~10 nl droplets with a fluorogenic enzyme substrate using an appropriate spore to droplet ratio allowing compartmentalization of single spores. 3. The emulsion is incubated 24 h off-chip at 30 °C in a glass capillary to allow fungi development, enzyme secretion and substrate digestion within the droplets. 4. Droplets are reloaded into a sorting device and sorted based on enzymatic activity according to fluorescence intensity. 5. Fungi are recovered from sorted droplets and either characterized or subjected to another round of mutagenesis and/or selection. (**b**) *α*-amylase fluorogenic assay. The substrate consists of a starch backbone with multiple quenched BODIPY^®^FL fluorophores. *α*-amylase hydrolyzes the starch backbone to unquench the fluorophores and induce fluorescence.

The screening platform for *Aspergillus niger* (Fig. 3a) was composed of two distinct microfluidic devices (Supplementary Fig. S1). A drop maker allowed the encapsulation of single spores with a fluorogenic *α*-amylase substrate (Supplementary Movie S2). Droplets of 18 nl were produced at ~80 Hz by hydrodynamic flow focusing of a spore suspension with a fluorinated oil phase containing 2.5% (w/w) KryJeffD_900_ fluorosurfactant. Fluorinated oils have been shown to facilitate respiratory gas delivery to both prokaryotic and eukaryotic cells in culture^27^. The number of spores per droplet follows a Poisson distribution^28^ and was controlled by adjusting the initial density of the spore suspension to give an average number of spores per drop *λ*, of 0.1–0.3. The droplets were collected in a glass capillary and incubated for 24 h at a 30 °C to allow germination, hyphal growth and *α*-amylase secretion within the droplets. After incubation, the droplets were re-injected into a sorting device with cross-side configuration electrodes (Supplementary Fig. S1 and Movie S7) which was used to sort droplets based on *α*-amylase activity (Supplementary Movie S8). The fluorogenic substrate was starch labeled with multiple BODIPY^®^FL fluorophores which are auto-quenched until the starch is hydrolyzed, resulting in an increase in green fluorescence (Fig. 3b). Sorted fungi were recovered, sporulated and further characterized.

#### 

**Figure 4:**
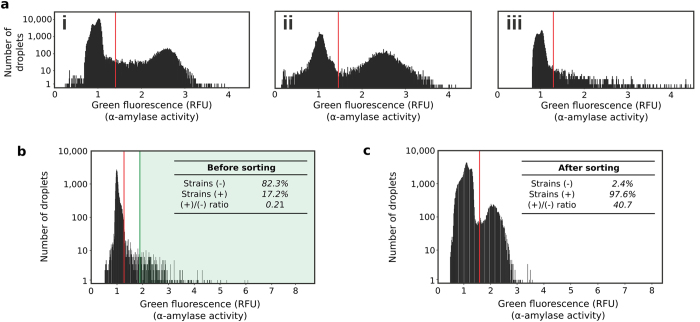
Screening *Aspergillus niger* libraries for *α*-amylase secretion. (**a**) Phenotype distribution (*α*-amylase activity) within large fungi populations. Histograms of the green fluorescence signal (514 nm; corresponding to *α*-amylase activity) of the droplets after incubation at 30 °C for 24 h of the O58 strain (i), a chemically mutated library (2 h exposure) (ii) or a UV-mutated library (60 s exposure) (iii). The wild-type histogram is displaying ~200,000 droplets (~20,000 fungi). The library histograms are each displaying ~30,000 droplets (~6,000 fungi). (**b**) Histogram of the green fluorescence signal (514 nm; related to *α*-amylase activity) of the droplets before sorting. To maximize enrichment for active variants, droplets were sorted (green region) only if green fluorescence was at least twice the modal fluorescence of the main population (empty droplets) i.e. >1.9 RFU. (**c**) Histogram of the green fluorescence signal (514 nm; related to *α*-amylase activity) of the selected fungi population. For all histograms, if *σ* is the standard deviation of the green fluorescence of the main population (empty droplets), droplets are considered (+) if RFU >3*σ* and (−) if RFU ≤3*σ* (red line threshold). The number of droplets is converted into the number of strains based on the occupancy.

The microfluidic platform was applied to screen for *Aspergillus niger* strains which overproduce *α*-amylase from large whole-genome mutated libraries. The *Aspergillus niger* strain O58, a Soufflet *α*-amylase producing reference strain was exposed either to a chemical agent (2% N-Methyl-N′-Nitro-N-Nitrosoguanidine [MNNG]) or to UV light (254 nm, 1.30 mW.cm^−2^) to introduce mutations in the entire genome. The diversity of chemical and UV libraries was 9.10^6^ and 5.10^4^, respectively. The *α*-amylase activity of the O58 strain, the chemical library (2 and 3 h exposure) and the UV library (30, 60 and 90 s exposure) were analysed with the microfluidic platform (Fig. 4a and Supplementary Fig. S4). The phenotypic signature of the O58 strain presents an active population [6.2% of drops (theoretically 9.5% with *λ* = 0.1); 2.5 ± 17.6% RFU] well separated from empty drops or drops showing no *α*-amylase activity [93.8% of drops; 1 ± 7% RFU] (Fig. 4a). Exposure to MNNG for 2 h resulted in an only slightly modified phenotypic signature [23% of active drops (theoretically 19.7% with *λ* = 0.22); 2.42 ± 15.7% RFU] (Fig. 4a). In contrast, the phenotypic signature was heavily impacted by 60 s UV-exposure, and the library predominantly comprised variants exhibiting no or low *α*-amylase activity and only a small number of variants with phenotypes giving equal or higher green fluorescence (*α*-amylase activity) than the O58 strain [2.6% of active drops (theoretically 19.7% with *λ* = 0.22); 1.93 ± 50.3% RFU] (Fig. 4a).

Based on these results, the UV mutagenesis method (60 s exposure) was chosen for the subsequent screening steps. The high mutation rates with UV mutagenesis result in high mortality (60 s UV exposure results in a survival rate of 2.6% (Supplementary Fig. S4)) and a large number of inactive clones. However, a large fraction of inactive clones is also statistically associated, in a random whole-genome mutagenesis strategy, to a higher probability of targeting the desired pathway or gene.

A library was constructed using UV-mutagenesis (60 s exposure) of the O58 mother strain. The library was encapsulated in 18 nl droplets with the fluorogenic *α*-amylase substrate. The number of viable spores per droplets (21% occupancy; *λ* = 0.24) was controlled by adjusting the spore density based on the survival curve (Supplementary Fig. S4). After 24 h incubation at 30 °C, droplets were sorted at 10 Hz based on fluorescence, selecting droplets containing *α*-amylase producing fungi (Fig. 4b; green region) (Movie S8). Around 5.10^4^ droplets (~10^4^ fungi) were screened in about 90 min, 1.45% were sorted (~750 droplets) and 616 fungi were recovered on an agar plate. The enrichment of the screening steps was evaluated by analyzing sorted fungi at the single clone level (~7,000 fungi) (Fig. 4c). The activity distributions show an important (196-fold) enrichment for active fungi: more than 97% of sorted fungi showed *α*-amylase activity, the enrichment being limited by co-encapsulation events during the emulsification step^12^.

### Microtiter-plate and shake-flask fermentation assays

**Figure 5:**
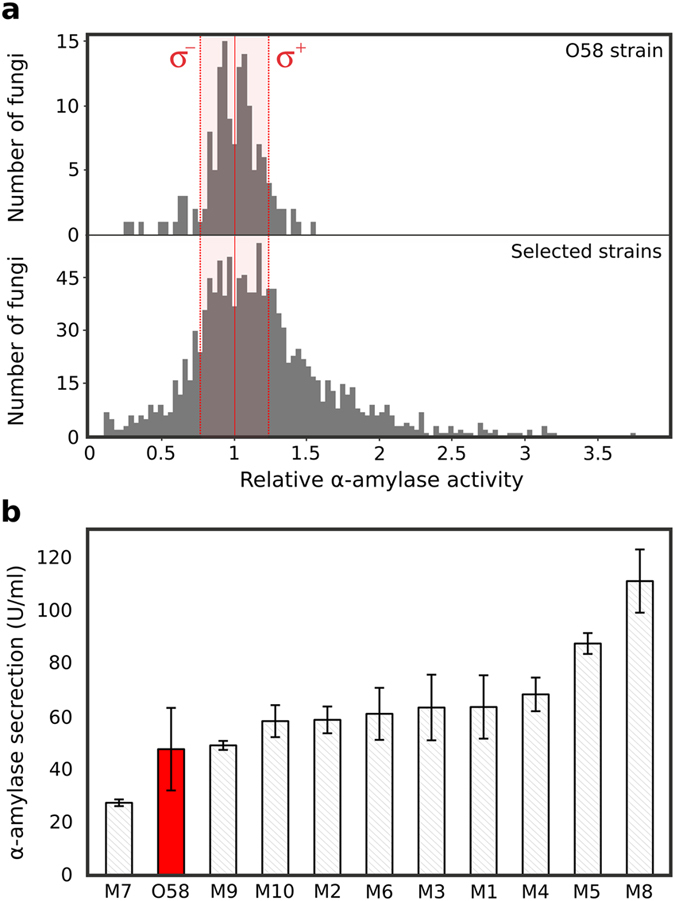
Microtiter-plate and shake-flask fermentation assays. (**a**) Relative *α*-amylase activity of the O58 strain (144 replicates) and the strains selected using the microfluidic screening (1,272 clones), analyzed using a robotic microtitre plate screening system. The standard deviation *σ* of the O58 strain is indicated. (**b**) Histogram displaying the *α*-amylase secretion level of 10 of the most active fungi from the plate screen and the wild type O58 strain after 6 days of cultivation at 30 °C in 100 ml of PGS medium. Mutants (gray) and O58 (red) were ranked from the lowest to the highest *α*-amylase activity. All fermentations were performed in duplicate and error bars correspond to ±1 standard deviation.

The sorted fungi population was screened using a microtitre plate-based robotic platform on an industrial relevant solid medium based on rapeseed meal. 1296 colonies were picked into 24-well plates, grown for 5 days and secreted *α*-amylase activity was measured in 96-well plates using the BODIPY^®^FL based substrate (Fig. 5a and Supplementary Fig. S5). 144 replicates of the O58 fungi were screened as positive controls. In total, 1,272 sorted fungi were analysed (87.2% of the sorted population): 98.9% were founded to be active while only 1.9% did not show detectable *α*-amylase activity. Most of the sorted fungi showed an activity level similar to O58 (47.3%) while 37.3% showed a higher activity (>1*σ*_*O*58_). We measured the skewness of each distribution as the Pearson coefficient of skewness. We obtained a value of −0.5 for the O58 distribution, meaning that the distribution is not significantly skewed compared to a normal distribution (p = 0.0124; *α* = 0.01), and a value of 1.05 for the sorted fungi distribution, indicating that the distribution has a significant positive skew (p < 0.00001; *α* = 0.01) (Supplementary Information).

We analysed in replicate 10 strains from the plate screen with *α*-amylase activity ≥3 by larger scale shake-flask fermentation. This format is closer to industrially relevant conditions and normally precedes further evaluation and scale-up in fermenters. The *α*-amylase activity was measured after 6 days of fermentation in PGS medium (100 ml) and compared to the O58 strain activity (Fig. 5b). All the strains tested except one displayed activity similar to or higher than O58, the best (M8) being 2.3-fold higher than the parental strain. The activities measured did not correlate exactly with those from microtitre plates, however, probably due the different production conditions (solid-state fermentation on rapeseed meal *versus* shake-flask fermentation in liquid synthetic PGS medium).

## Discussion

We have developped and applied an efficient new approach for the high-throughput screening of filamentous fungi based on secreted enzyme activities. Using droplet-based microfluidic tools adapted to manipulate nanoliter-volume droplets, the technique allows fast enrichment of large population of fungi and can be integrated to industrial screening processes to speed up the discovery of new production strains of biotechnological interest.

Compared to automated colony pickers and liquid handling systems, the microfluidic platform offers higher throughputs, lower reagent consumption and reduced spatial footprint, all of which contribute to lower screening costs (Supplementary Table S1): screening 10^4^ *A. niger* variants directly using the robotic microtitre plate-based screening platform would have taken more than 16 days and cost $8,770, whereas screening 10^4^ variants using the microfluidic system took less than 24 h and cost $14 (and the subsequent screening of 1,272 sorted variants using the microtitre plate-based system took only 8 days and cost $1,100), costs being estimated based on consumables only. In addition to inherent throughput and scale advantages related to the microfluidics format, this approach also greatly simplifies the manipulation of filamentous fungi: off-chip fungi handling is limited to the manipulation of spores and operations on mycelia are performed by manipulating droplets.

Combining microfluidic and robotic microtitre-plate HTS is an attractive strategy. Here, single fungal spores were compartmentalized in droplets together with a fluorogenic *α*-amylase substrate, and the droplets and the germinated fungi they contain were sorted after 24 h incubation, triggered on fluorescence. However, with such an endpoint measurement, it is difficult to distinguish reliably between variants exhibiting different levels of *α*-amylase activity as fungal growth, enzyme secretion and the fluorogenic *α*-amylase reaction are coupled, and in many droplets all (or most) of the substrate was consumed. However, sorting all variants with a fluorescent signal equal to or greater than the mother strain using the microfluidic system efficiently removed variants showing reduced or no *α*-amylase activity: before sorting, 83% of clones showed little or no *α*-amylase activity, but a single round of microfluidic sorting resulted in a 196-fold enrichment of active clones. The throughput of microfluidic HTS enables the screening of highly mutated libraries, which are mainly composed of inactive clones, but where there is also a higher probability of finding beneficial mutations, as the higher mutation rate increases the chance of hitting the target gene(s).

Furthermore, the microfluidic assay could be made more quantitative by uncoupling fungal germination, growth and enzyme secretion from the enzymatic assay. This could be achieved by adding the fluorogenic substrate to droplets after incubation to allow fungal growth and enzyme secretion using droplet electrocoalescence^29^ or picoinjection^30^. It may also be possible to increase the throughput above ~7,000 fungi.h^−1^. The maximum experimental sorting rate (21 Hz) is close to the calculated theoretical maximum sorting rate limited by DEP and electrocoalescence (46 Hz). However, the sorting frequency was not limited by DEP forces or electrosplitting, but by purely fluidic constraints: higher reinjection frequencies resulted in droplets breaking when oil was added to space the reinjected droplets. Higher sorting rates could potentially be achieved by reducing the oil spacer flow rate, a strategy which, combined with other sorter design improvements, allowed sorting of pl volume droplets at 30 kHz^31^. Throughput could be further increased, at least to a certain extent, by increasing *λ* (at the expense of increasing the number of false positives due to co-compartmentalization of more than one variant in the same droplet^12^).

The microfluidic system is not limited to screening for *α*-amylase activity: filamentous fungi can potentially be screened for the production of other enzymes or metabolites. The only restriction is that there must be a fluorogenic assay for the desired enzymatic activity or metabolite, and the metabolite and fluorescent product must not exchange between droplets over the time of the experiment. The use of fluorinated oils for the continuous phase mitigates against exchange, since non-fluorinated molecules are highly insoluble in fluorinated oils^32^. However, exchange can also be mediated by micellar transport^33^. Nevertheless, many microtitre-plate-based assays can be transposed directly to droplets, as was the case with the *α*-amylase assay used here, and direct, or coupled enzymatic assays can be used. Many other fluorogenic assays can also be simply adapted for microfluidic systems by chemically modifying the fluorogenic substrate to increase the hydrophilicity of the fluorescent product^34^, which reduces partitioning into micelles in the continuous phase and exchange between droplets^33^. For example, droplet-based microfluidic assays have been developed to screen for bacteria and yeast producing cellulases, based on either directly screening for exogluconase (cellobiohydrolase) activity using a fluorogenic assay^35^ or using a coupled fluorogenic enzyme assays to measure endogluconase activity^16^, and have been used for the ultrahigh-thoughput bioprospection of natural cellulolytic bacteria^35^. In the future, novel assays based on, for example, changes in osmotic pressure^36^,^37^ or mass spectroscopy^38^,^39^,^40^ might also be developed.

The ability to screen large filamentous fungi populations in a few hours at low cost should bring enormous benefit for identification of new biotechnologically interesting filamentous fungi. Besides this promising biotechnological application, this screening platform would also be applicable to more fundamental studies in combination with whole genome sequencing to help identify mutations and thereby regulatory networks responsible for improved phenotypes. The understanding of those genetic factors might be used to optimize filamentous fungi via inverse metabolic engineering^41^.

## Methods

### Fabrication of microfluidic devices

Poly-(dimethylsiloxane) (PDMS) microfluidic devices were fabricated as previously described^42^ from 250 *μ*m-deep molds of SU-8 2150 negative photoresist (MicroChem Corp).

### Optical setup, data acquisition and control system for droplet analysis

The optical setup used to monitor microfluidics experiments was previously described, as well as the data acquisition and control system^34^. In addition, to allow the sorting of a particular droplet, the data acquisition card provided a signal to a model 623B high-voltage amplifier (Trek Inc.) connected to the electrodes of the microfluidic device.

### Surfactant synthesis

We used aqueous droplets in Novec7500 fluorinated oil (3M) stabilized against coalescence by a triblock copolymer fluorosurfactant comprising two perfluoropolyether (PFPE) chains linked by one Jeffamine^®^ polyetheramine chain (PEA), KryJeffD_900_. KryJeffD_900_ surfactant was prepared in house from the commercially available carboxylic acid Krytox157-FSH (Dupont) and Jeffamine^®^ polyetheramine (ED 900, Huntsmann) based on the synthesis route described in^23^,^43^. Briefly, Krytox157-FSH (50 g; 7.8 mmol assuming 6500 g.mol^−1^) was dissolved in 150 ml of CaCl_2_ dried Novec7100 oil under a N_2_ atmosphere. Next, oxalyl chloride (7.7 ml; 90 mmol) was added dropwise and the mixture stirred overnight at 70 °C. After evaporating the solvent, the resulting product was dissolved in 100 ml of FC-3283 oil (3M). Jeffamine^®^ polyetheramine (3.3 ml; 7.9 mmol), 20 ml of dried tetrahydrofuran, thriethylamine (1.6 ml; 11.5 mmol) and 40 ml of dried tetrahydrofuran were subsequently added in a twin-neck round-bottom flask under a N_2_ atmosphere. The Krytox157-FSH acid chloride solution was added in the flask, followed by 20 ml of FC-3283 oil (3M). The mixture was stirred overnight at room temperature. After removing the solvents and purification by filtration, the product was used directly in the experiments.

### Microfluidic chip operation

Liquids were pumped into the microfluidic devices using standard-pressure infusion-only PHD 22/2000 syringe pumps (Harvard Apparatus Inc.). Syringes (Omnifix-F^®^ BBRAUN) were connected to the microfluidic devices using 1.2 × 40 mm needle (Terumo) and PTFE tubing (Fisher Scientific) with an internal diameter (ID) of 1.06 mm and an external diameter (OD) of 1.68 mm. Droplets were produced using a dropmaker device (Supplementary Fig. S1) by flow-focusing of the aqueous stream with two streams of Novec7500 oil containing 2.5% (w/w) of KryJeffD_900_ surfactant. The device was used to produce 10 nl droplets (Q_*aqueous*_ 3 ml.h^−1^, Q_*oil*_ 10 ml.h^−1^, 80 droplets.s^−1^), 18 nl (Q_*aqueous*_ 6 ml.h^−1^, Q_*oil*_ 8 ml.h^−1^, 90 droplets.s^−1^) or 20 nl droplets (Q_*aqueous*_ 4.6 ml.h^−1^, Q_*oil*_ 5 ml.h^−1^, 90 droplets.s^−1^) depending on the experiment. The generated emulsions flowed off-chip through PTFE tubing to a glass capillary attached to a Peltier device for off-chip incubation^44^. Droplets were reloaded (Q_*droplets*_ 125–700 *μ*l.h^−1^) in a fluorescence activated droplet sorting (FADS) device (Supplementary Fig. S1) and spaced with Novec7500 oil (Q_*oil*_ 10–21 ml.h^−1^). The droplets were analysed by the optical setup and fluorescent droplets were sorted at 4 to 20 droplet.s^−1^ by applying AC field pulses (30 kHz; 1400–1800 V_*pp*_; 10–40 ms). The two collection outlets were connected to TYGON tubing (7.94 mm OD; 4.76 mm ID) via 6 mm-diameter L-shaped connectors (VWR).

### Fluorescence-activated droplet sorting validation

A binary emulsion comprising 10 nl droplets was produced by mixing two emulsions produced successively using the dropmaker device. The first droplet population contained 1 *μ*M of sulforhodamine B in YGC medium (Yeast extract 5 g.l^−1^, Glucose 20 g.l^−1^, Chloramphenicol 0.1 g.l^−1^) and the second contained 30 *μ*M of sulforhodamine B and blue ink colorant in YGC medium. The binary emulsion was reloaded into the sorting device and sorted as a function of red fluorescence to select only those droplets containing 30 *μ*M of sulforhodamine B. Both sorted and unsorted droplets were collected. The performance of the sorter was evaluated before and after sorting either by imaging the emulsion on a glass slide using a color camera (Nikon DS-Fi2) or by video analysis of the sorting process using a high-speed camera (Mikroton Eosens MC1310).

### Filamentous fungi strain and culture conditions

The *Aspergillus niger* O58 strain was obtained from the Ets J. Soufflet strain collection. The O58 strain was plated on a PDA (AES Laboratoire) plate and grown at 30 °C for 10 to 15 days for optimal sporulation. Spores were suspended in 20 ml of PGS medium (Glucose 10 g.l^−1^, Pancreatic peptone 6 g.l^−1^, MgSO_4_.7H_2_O 0.5 g.l^−1^, KH_2_PO_4_ 0.5 g.l^−1^, FeSO_4_.7H_2_O 0.5 mg.l^−1^). The spore suspension was stirred for 10 min, filtered through sterilized gauze to remove mycelium residues and stirred again for 10 min. The spore density was estimated using a Thoma cell counting chamber.

### UV mutation

After sporulation, spores were suspended in 0.01% Triton X-100 (20 ml). The spore suspension was stirred for 10 min, filtered through sterilized gauze and stirred again for 10 min with glass beads to homogenize. The spore concentration was adjusted to 3.10^6^ spores per ml. 3 ml of the spore suspension was spread on a glass petri dish and exposed to 6 W UV light (Dymax PC-2000/38003) at 254 nm for 60 s at a distance of 5 cm from the lamp (1.30 mW.cm^−2^) to introduce mutations randomly throughout the whole fungi genome. The procedure was repeated three times to UV treat a total of 9 ml of spore suspension. The UV-treated spore suspension was centrifuged 10 min at 2500 g and the supernatant was discarded. The pellet was suspended in PGS medium.

### MNNG mutation

As for UV mutation, a spore suspension was prepared. The spore concentration was adjusted to 1.10^7^ spores per ml. 5 ml of 0.2 M phosphate buffer (pH 6.2) containing 6% (w/w) of N-Methyl-N′-Nitro-N-Nitrosoguanidine (MNNG) was added to 10 ml of spore suspension. The suspension was stirred at 30 °C for the desired mutagenesis time. Mutated spores were then centrifuged 5 min at 4500 g and the pellet was suspended in 5 ml of Triton X-100 (1 g.l^−1^). The procedure was repeated three times to wash away the mutagenesis agent. The pellet was finally suspended in PGS medium.

### Characterization of *α*-amylase activity of O58 strain and libraries

The spore suspension was diluted to the appropriate spore to droplet ratio to obtain typically 0.1–0.2 spore per droplet in PGS medium containing the BODIPY^®^FL-labeled DQ^*TM*^ starch substrate (50 *μ*g.ml^−1^; Enzchek Ultra Amylase Assay Kit; Life Technologies) and Sulforhodamine 101 (5 *μ*M). Spores were encapsulated in 18 nl droplets using the dropmaker device. The emulsion was collected in a glass capillary, incubated off-chip for 24 h at 30 °C and then reloaded in a FADS device for fluorescence analysis.

### Microfluidic screening of libraries for *α*-amylase production

The library spore suspension was encapsulated in 18 nl droplets as described in the previous paragraph. The emulsion was collected in a glass capillary, incubated off-chip for 24 h at 30 °C and then reloaded in a FADS device for fluorescence analysis and selection of droplets containing active fungus at 10 droplets.s^−1^ by applying AC field pulses (30 kHz; 1600 V_*pp*_; 35 ms). Sorted fungi were spread on PDA plates, grown for 48 h at 30 °C, counted, and then grown for an additional 6 days to allow sporulation. For conservation, spores were suspended in 20 ml of water, counted on a Thoma cell counting chamber and frozen at −80 °C in 15% glycerol at a density of at least 10^6^ spores per ml.

### Microplate-based screening

The sorted fungi population and O58 strain were grown on agar plates to feed a Hamilton Microlab STAR 8/96 ML picking robot. Spores were plated on PDA plates (30 plates for the O58 strain and 150 plates for the sorted population) and grown for 40 h at 30 °C to give 15 to 20 thalli per plate. The picking robot was run for 3 days to transfer every strain into 24-wells plates containing agar growth medium based on rapeseed meal (rapeseed meal 100 g.l^−1^; pancreatic peptone 6 g.l^−1^; MgSO_4_ 7 H_2_O 0.5 g.l^−1^; KH_2_PO_4_ 0.5 g.l^−1^; FeSO_4_ 7H_2_O 0.5 mg.l^−1^, Agar 20 g.l^−1^). Plates were incubated for 5 days at 30 °C, 95% hygrometry. 1 ml of water was then added to each well and plates were incubated 48 h at 4 °C for protein extraction. Aqueous extracts were transferred in 24-well plates for automated titration of *α*-amylase activity using a Hamilton Microlab STARlet 4 ML liquid handling robot. The fluorescence read out was performed in 96-wells plates over 10 min at 45 °C from 25 *μ*l of sample added to 25 *μ*l of BODIPY^®^FL-labeled DQ^*TM*^ starch substrate (50 *μ*g.ml^−1^; Enzchek Ultra Amylase Assay Kit; Life Technologies; *λ*ex 480 nm; *λ*em 515 nm) in citrate buffer (0.33M, pH3.3). A calibration curve was produced using an *α*-amylase standard (7500 U.g^−1^) for the 0–300 mU.ml^−1^ range (Supplementary Fig. S5).

### *α*-amylase secretion in shake-flask fermentation

For each strain, two 2.5 l flasks containing 100 ml of PGS medium were each inoculated with 1 ml of a glycerol stock spore suspension (5.10^8^ spores.ml^−1^). The flasks were incubated at 30 °C (150 rpm) for 6 days. Supernatants were titrated for *α*-amylase activity. The fluorescence readout was performed in 96-wells plates over 10 min at 45 °C from 25 *μ*l of sample added to 25 *μ*l of BODIPY^®^FL-labeled DQ^*TM*^ starch substrate (50 *μ*g.ml^−1^; Enzchek Ultra Amylase Assay Kit; Life Technologies; *λ*ex 480nm; *λ*em 515 nm) in citrate buffer (0.33 M, pH3.3).

## Additional Information

**How to cite this article**: Beneyton, T. *et al.* High-throughput screening of filamentous fungi using nanoliter-range droplet-based microfluidics. *Sci. Rep.* **6**, 27223; doi: 10.1038/srep27223 (2016).

## Supplementary Material

Supplementary Information

Supplementary Information

Supplementary Movie 1

Supplementary Movie 2

Supplementary Movie 3

Supplementary Movie 4

Supplementary Movie 5

Supplementary Movie 6

Supplementary Movie 7

Supplementary Movie 8
