# Supplementary material for: High-throughput screening of filamentous fungi using nanoliter-range droplet-based microfluidics
Source: Sci Rep. 2016 Jun 7;6:27223. doi: 10.1038/srep27223 (PMC4895158; doi:10.1038/srep27223)
Supplement: Supplementary Information [file srep27223-s1.pdf]

High-throughput screening of filamentous fungi using nanoliter-range droplet-based microfluidics

Thomas Beneyton[1,+], I Putu Mahendra Wijaya[1,2,+], Prexilia Postros[2], Majdi Najah[2], Pascal Leblond[2], Angélique Couvent[2], Estelle Mayot[2], Andrew D. Griffiths[1,*], and Antoine Drevelle[2]

[1]École Supérieure de Physique et de Chimie Industrielles de la Ville de Paris (ESPCI ParisTech), CNRS UMR 8231, 10, rue Vauquelin, 75231 Paris Cedex 05, France.
[2]Ets J. Soufflet/CRIS-OSIRIS, Quai Sarrail, BP12, 10400 Nogent-sur-Seine, France
[*]andrew.griffiths@espci.fr
[+]these authors contributed equally to this work

Supplementary Information

Supplementary Figure S1

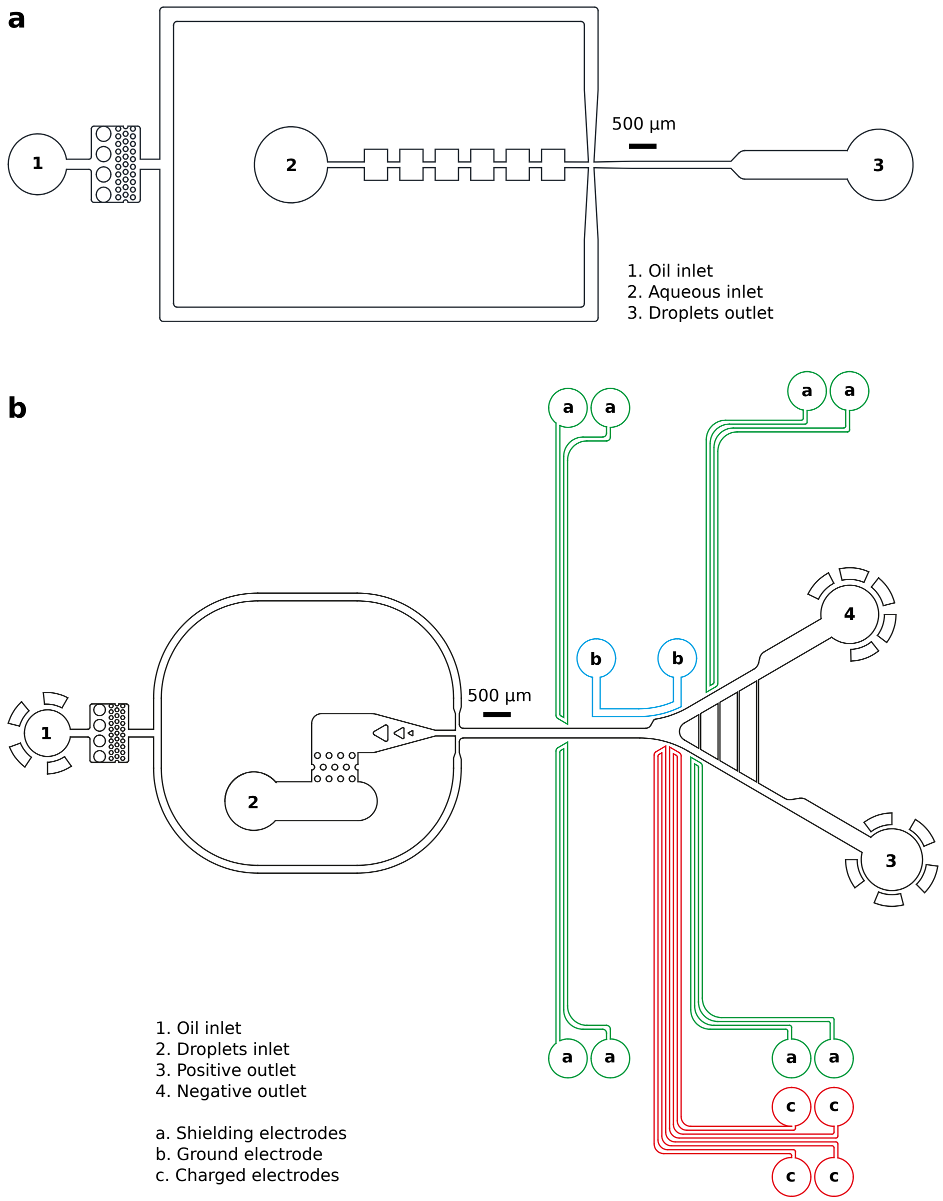

Figure S 1. Description of microfluidic modules. (a) Dropmaker device: droplets are produced by flow-focusing of the aqueous stream[1] with two streams of fluorinated oil containing surfactant. The device was used to produce 10, 18 or 20 nl droplets depending on the experiment. (b) Fluorescence Activated Droplet Sorter (FADS) device: droplets are reloaded (2) and spaced-out at a flow-focusing junction with fluorinated oil. The droplets are analyzed by the optical setup and fluorescent droplets are sorted at 4 to 20 droplet.s^{-1} by applying AC field pulses (30 kHz; 1400-1800 V_{pp}; 10 - 30 ms).

Supplementary Figure S2

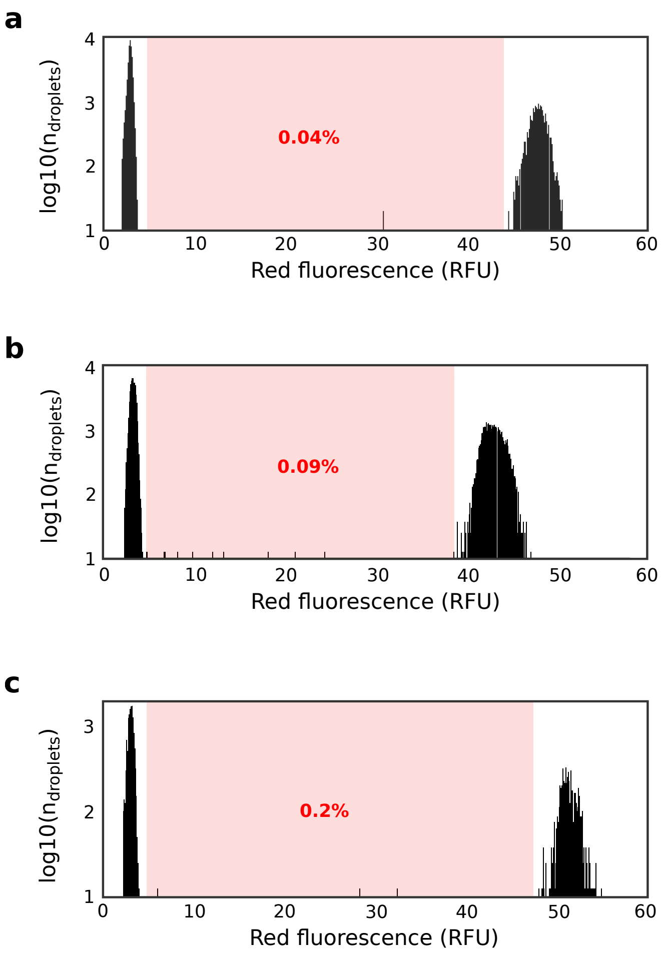

Figure S 2. Emulsion stability. (a-c) Fluorescence histograms for three emulsions comprising a 1:1 mixture of 10 nl droplet containing 1 μM or 30 μM sulforhodamine B, respectively after 24 h incubaton at 30°C. Droplets were reloaded in the FADS device for fluorescence analysis. The droplets with intermediate fluorescence (red zone) corresponding to coalesced droplets is shown in red text.

Supplementary Figure S3

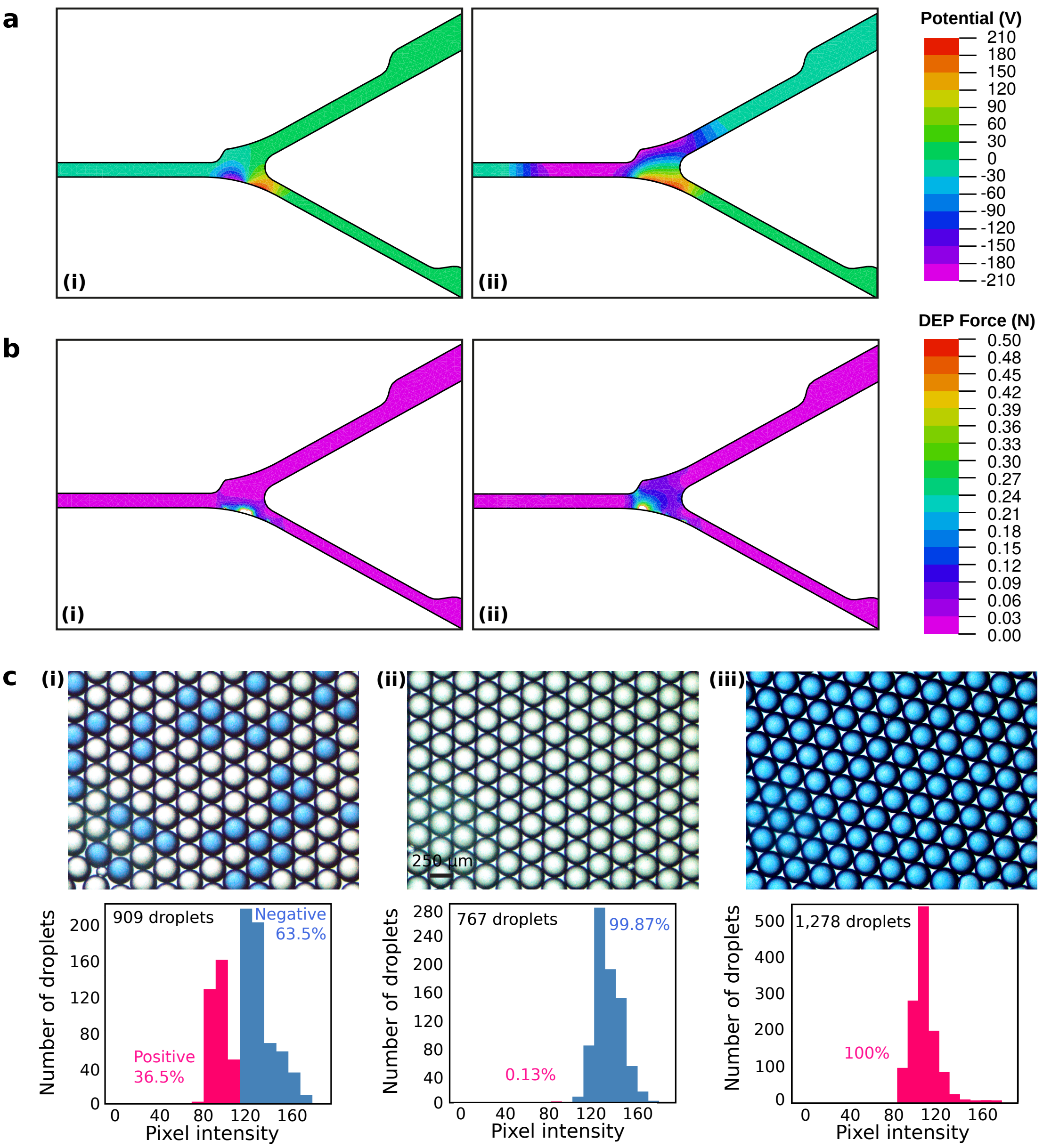

d

Droplet volume	Sorting rate	n droplets	n unsorted	n sorted	n flase +	n flase -	Electric field parameters Amplitude	Frequency	Pulse time	Flow rates (ml.h^{-1}) Emulsion	Oil
10 nl	4 Hz	1,466	964	501 (34.2%)	0 (<0.07%)	1 (0.07%)	1,400 V	10 kHz	30 ms	0.125	10
	12 Hz	131	84	46 (35.1%)	0 (<0.76%)	1 (0.76%)	1,600 V	10 kHz	10 ms	0.4	17
	17 Hz	211	126	85 (40.3%)	0 (<0.47%)	0 (<0.47%)	1,700 V	10 kHz	10 ms	0.55	19
	21 Hz	113	70	39 (34.5%)	0 (<0.88%)	4 (3.5%)	1,800 V	10 kHz	10 ms	0.7	21
20 nl	4 Hz	352	209	133 (37.8%)	0 (<0.28%)	10 (2.8%)	1400 V	10 kHz	35 ms	0.15	8

Figure S 3. Validation of the FADS module. (a-b) Finite-element simulations of potential (a) and dielectrophoresis force (DEP) (b) distributions across the FADS device channels with the same-side electrode configuration (i) or the cross-side electrode configuration (ii). (c) Color image analysis of the sorting efficiency. Images of the emulsion before sorting (i), of unsorted (ii) and sorted (iii) droplets were analyzed using a homemade MATLAB R2013a routine to distinguish and count negative colorless droplets and positive blue droplets. (d) Evaluation of the efficiency of the sorting process. Movies of the sorting process were analyzed to determine the number of false negatives and false positives using different throughputs and droplet volumes. The table summarizes the results of the video analysis, as well as the electric field parameters and the flow rates of the emulsion and the spacing oil.

Supplementary Figure S4

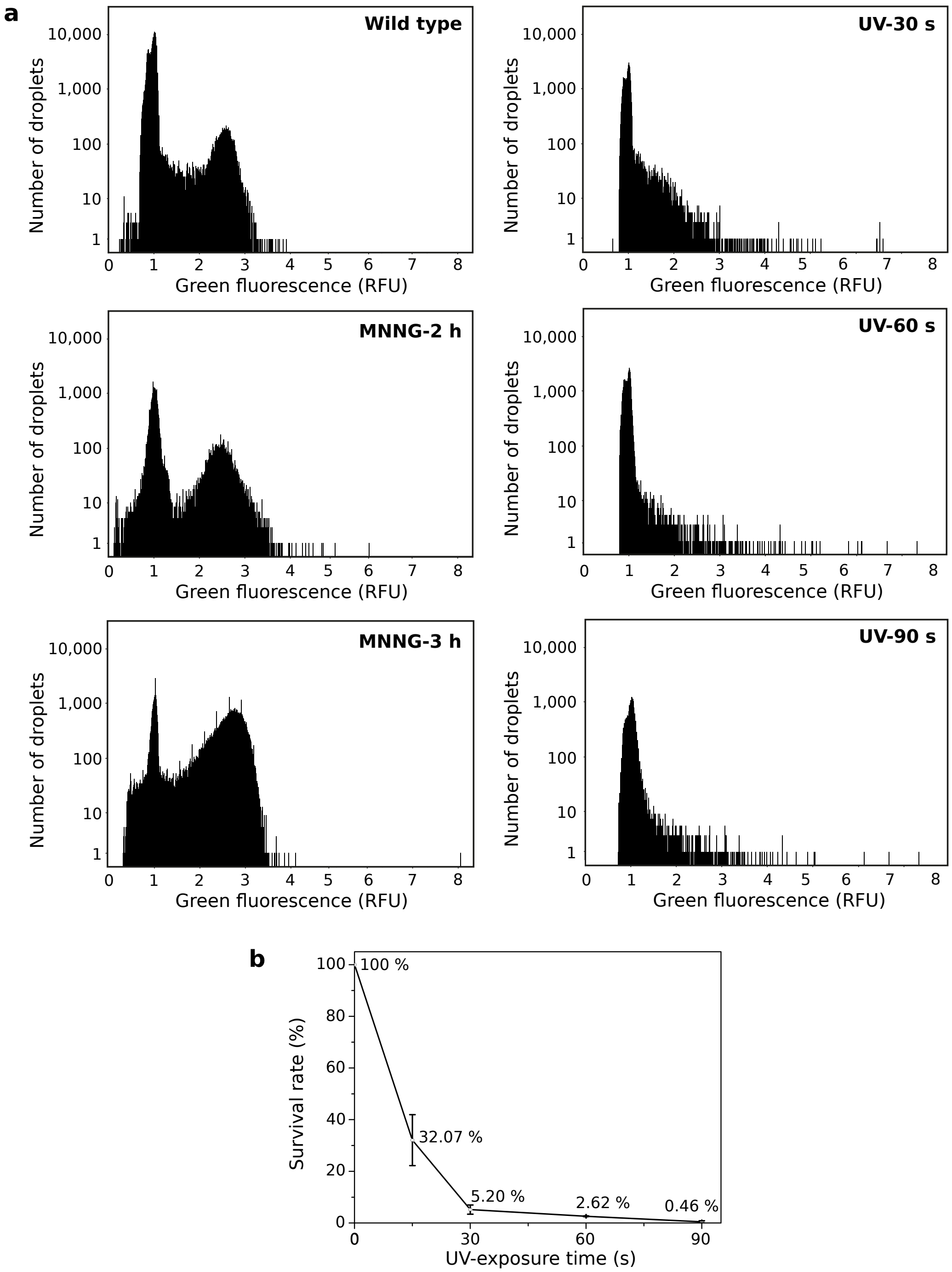

Figure S 4. Library construction. (a) Phenotype distribution (α-amylase activity) within different libraries measured at the single individual level using droplet-based microfluidics. Histograms of the green fluorescence signal (514 nm; related to α-amylase activity) of the droplets after single spore encapsulation with the fluorogenic substrate and incubation at 30°C during 24 h in the cases of the O58 wild type strain, chemical treatment (2 or 3 h exposure to 2% MNNG) or UV treatment (30, 60 or 90s exposure to [254 nm, 1.30 mW/cm^{-2})] light). The wild-type histogram shows data from ~200,000 droplets (~20,000 clones) while the histograms of the libraries each show data from ~30,000 droplets (~6,000 strains). (b) Survival curve of *Aspergillus niger* O58 strain exposed to or UV light (254 nm; 1.30 mW.cm^{-2}). Error bars represent ±1 standard deviation based on triplicate measurements.

Supplementary Figure S5

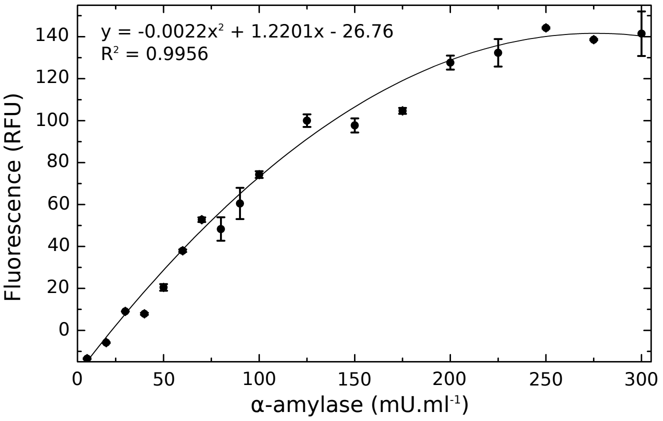

Figure S 5. Microplate α-amylase titration. A calibration curve was produced using an α-amylase standard (7500 U.g^{-1}) for the 0–300 mU.ml^{-1} range. The α-amylase activity was measured using the BODIPY®FL-labeled DQ^{TM} starch assay in microtiter plate at 45°C (λex = 480 nm; λem = 515 nm) in citrate buffer (0.33M; pH3.3). Error bars represent the standard deviation based on triplicate measurements.

Supplementary Figure S6

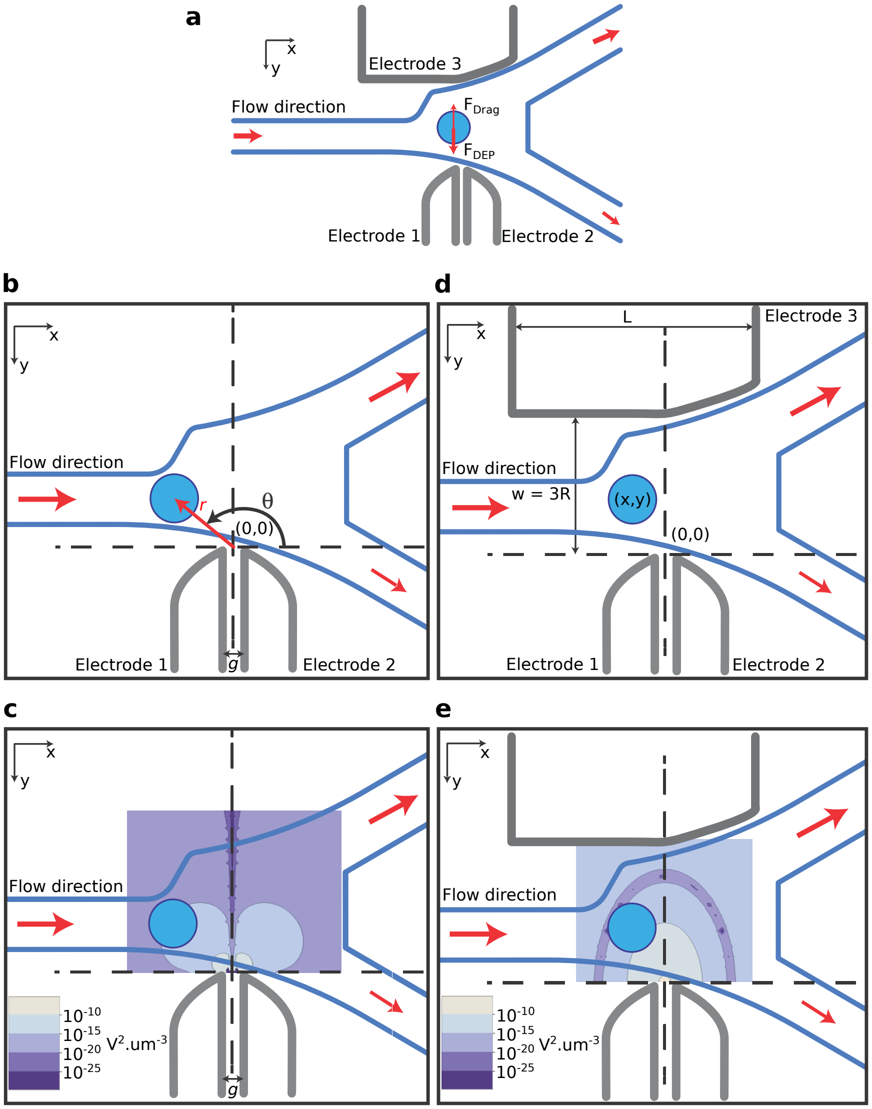

Figure S 6. Same-side vs. cross-side electrode configuration. (a) Force diagram of a droplet passing through a sorting chamber. The outlet channels have different widths. By default, droplets flow into the wider channel due to its lower fluidic resistance. When an electric bias is applied to the electrodes, however, the DEP force (F_{DEP}) displaces the droplet in the y-axis orthogonal to the flow, directing the droplets into the narrower channel. The fluorinated carrier oil around the droplet imparts drag force (F_{Drag}) that opposes the DEP force. The interplay between these two forces determines the sorting frequency of the droplets. In the same-side configuration, electrode 1 and electrode 2 are connected to the positive and negative outlets of the high voltage amplifier, respectively, and electrode 3 is left unconnected. In the cross-side configuration, electrode 1 and electrode 2 are connected to the positive outlet from the high-voltage amplifier, while electrode 3 is connected to the negative outlet. (b) Setup for modeling the same-side electrode configuration. The origin in the derivation is located at the mid-point between the tips of the two electrodes. The r represents the distance from the origin to the centroid of the droplet at any given point in time, while θ refers to the angle made between the x-axis and the line r, calculated in counter-clockwise fashion. The distance between the electrodes is g. (c) Distribution of field inhomogeneity $|\nabla|\vec{E}|^2|$ with the same-side electrode configuration. The distance between the electrodes is g. (d) Setup for modeling the cross-side electrode configuration. The analytical solution to the electric field distribution can be calculated using an approach based on a point and line electrode. Similar to the previous calculation, the origin in this derivation is located at the mid-point between the tips of electrode 1 and electrode 2. In the cross-side configuration, electrode 1 and electrode 2 are both connected to the positive outlet from the high-voltage amplifier, while electrode 3 is connected to the negative outlet from the high-voltage amplifier. This connection establishes a voltage bias across the sorting chamber. This is in contrast to the same-side configuration, in which the voltage difference is set only on one side of the sorting chamber. (e) Spatial distribution of field inhomogeneity $|\nabla|\vec{E}|^2|$ with the cross-side electrode configuration.

Supplementary Table S1

	Time footprint		Cost footprint	
	Microfluidics	*Microtiter-plate*	*Microfluidics*	*Microtiter-plate*
Compartmentalization	Emulsification<br>5.10^4 clones.h^{-1}<br>**<1 h**	Picking<br>50 clones.h^{-1}<br>**200 h**	Medium: 18 nl x 5.10^4 = 0.9 ml<br>**<$1**<br>Syringe, tubing, oil, device<br>**$1**	Medium: 1 ml x 10^4 = 10 l<br>**<$1**<br>24-well plates x420<br>**$420**
Culture	Overnight incubation<br>**15 h**	5 days incubation<br>**120 h**<br>Enzyme solubilization<br>**48 h**	-<br>-	Water: 1 ml x 10^4 = 10 l<br>**<$1**<br>Tips x10,000<br>**$500**
Screening	7,000 clones.h^{-1}<br>**1.5 h**	400 clones.h^{-1}<br>**25 h**	Fluorogenic substrate: 45 µg<br>**$12**<br>Syringe, tubing, oil, device<br>**$1**	Fluorogenic substrate: 25 mg<br>**$6,700**<br>96-well plates x105<br>**$150**<br>Tips x20,000<br>**$1,000**
**Total**	**<24 h**	**393 h**	**$14**	**$8,770**

Table S 1. Estimation and comparison of time and consumables cost footprints for the screening of 10^4 fungi using a traditional plate-based systems or the microfluidic system. Compared to plate-based system, the cost and time footprints of a microfluidics screen are more than 600 and 16 times lower, respectively. Combining a 10^4 fungi microfluidic screen with a subsequent plate-based screen of 1,272 fungi reduces the cost and time footprints by 8 and 2, respectively, compared to a full plate-based screen.

Supplementary Video S1

Movie S1. Spore germination and hyphal growth within 250 pl water in oil droplet. Single spores were encapsulated in 250 pl droplets containing PGS growth medium. Droplets were immobilized in a drop spot array and incubated for 17h at 30°C.

Supplementary Video S2

Movie S2. Production of 10 nl droplets using the dropmaker device.

Supplementary Video S3

Movie S3. Fluorescence-activated sorting of 10 nl droplets using (1.4 kV; 30 ms) AC pulses in the case of the cross-side electrode configuration

Supplementary Video S4

Movie S4. Fluorescence-activated sorting of 10 nl droplets using (1.4 kV; 30 ms) AC pulses in the case of the same-side electrode configuration

Supplementary Video S5

Movie S5. Fluorescence-activated sorting of 10 nl droplets using at 4 Hz.

Supplementary Video S6

Movie S6. Fluorescence-activated sorting of 10 nl droplets using at 17 Hz.

Supplementary Video S7

Movie S7. Reinjection of 10 nl droplets in the Fluorescence-Activated Droplet Sorter (FADS) device.

Supplementary Video S8

Movie S8. Sorting of *Aspergillus niger* fungi based on α-amylase secreted activity in 18 nl droplets.

Theoritical analysis of dielectrophoretic sorting

In dielectrophoretic droplet sorters,[1–3] droplets flow in carrier oil towards a Y-shaped junction. With no electric field, all drops flow into the waste channel which offers lower hydrodynamic resistance than the second, collect channel. To direct droplets into the collect channel, on chip electrodes are energized, creating an electrical field gradient, which generates a dielectrophoretic force (DEP) acting on the droplets. In order to be sorted, DEP forces must displace the droplet by a critical distance orthogonal to the flow, d_o, in the time, t_p, it takes the droplet to traverse the electrical field gradient. The DEP force on a spherical droplet is given by equation (1),

$$|\vec{F_{DEP}}| = 4\pi\tilde{\varepsilon}_m^* R^3 Re[f_{CM}]\nabla|\vec{E}|^2 \tag{1}$$

in which, $|F_{DEP}|$ is the magnitude of the DEP force, R is the radius of the droplet being subjected to the DEP force, $Re[f_{CM}]$ is the Claussius-Mosscoti factor (which itself depends on $\tilde{\varepsilon}_p^*$ and $\tilde{\varepsilon}_m^*$: the complex permittivity of the droplet and the carrier fluid, respectively) and $\nabla|\vec{E}|^2$ denotes the electrical field gradient. The carrier oil, however, imparts drag force, which opposes the DEP force as the droplet moves through the fluid orthogonal to the flow (Supplementary Fig. S6). In the limit of low Reynolds number, relevant in microfluidic environment, this drag force is described by:[1]

$$F_{drag} = 6\pi\eta R v \tag{2}$$

where η designates the viscosity of the carrier oil, R denotes the radius of the droplet, and v denotes the velocity of the droplet. Hence, applying Newton's second law, the following relationship can be obtained:

$$\sum F_y = 4\pi\varepsilon_m R^3 Re[f_{CM}]\nabla|\vec{E}|^2 - 6\pi\eta R v_y = m\frac{dv_y}{dt} \tag{3}$$

Equation (3) describes the movement of the droplet in the y-axis under the influence of DEP and the drag force. By solving equation (3), we can describe the velocity of the droplet in the y-axis as a function of time:

$$v_y = \frac{2R^2\varepsilon_m Re[f_{CM}]\nabla|\vec{E}|^2}{3\eta}(1 - e^{\frac{-6\pi\eta Rt}{m}}) \tag{4}$$

Equation (4) shows that the DEP causes the droplet to accelerate along the y-axis, orthogonal to the flow, before reaching a terminal velocity, $v_{t,y}$ when the drag force balances the DEP force (since F_{drag} increases with v):

$$v_{t,y} = \frac{2R^2\varepsilon_m Re[f_{CM}]\nabla|\vec{E}|^2}{3\eta} \tag{5}$$

Furthermore, equation (4) also shows the transition time, τ, to reach terminal velocity

$$\tau = \frac{m}{6\pi\eta R} \tag{6}$$

In the regime where the droplet is spherical, the mass of the droplet is related to its radius through relationship:

$$m = \rho\frac{6\pi R^3}{3} \tag{7}$$

Substituting equation (7) into equation (6), τ can be written as:

$$\tau = \frac{2\rho R^2}{9\eta} \tag{8}$$

Taking the typical values for all the variables used in the experiments (ρ= 1,000 kg.m^{-3}; R = 250.10^{-6} m; and η= 0.77 Pa.s) the transition time $\tau \approx$ 18 μs. This transition time is significantly smaller than the duration of the sorting pulse, (10 - 40

ms). Hence, the droplet can be assumed to be at terminal velocity throughout the sorting process and equation (5) can be used to describe the movement of the droplet in the y-axis within the sorting chamber. However, the droplet also travels in the x-axis, parallel to the flow, carried by the oil. If the droplets do not interact with each other mechanically, the terminal velocity of the droplet owing to pressure difference is relatively constant.[4] Hence, equation (5) can be used to estimate the theoretical maximum sorting frequency, f_{sort}. Assuming that the critical distance d_0 the droplet must be displaced in the y-axis in order to be sorted equals the diameter of the droplet: $d_0 = 2R$; and that in order to prevent false positives, no more than one droplet can be in the electric field in the sorting chamber at any one time:

$$f_{sort} = \frac{v_{t,y}}{w_{sort}} = \frac{v_{t,y}}{2R} = \frac{1}{2R}\frac{2R^2\varepsilon_m Re[f_{CM}]\nabla|\vec{E}|^2}{3\eta} \tag{9}$$

The derivation of Equation (9) assumes that as soon as one drop is sorted the next droplet arrives in the sorter. During our experiments, however, this was not the case. Droplets were spaced with sufficient carrier oil to prevent false positives due to two consecutive droplets being in the sorter at the same time ($Q_{droplets}$ 125-700 μl.h^{-1} and Q_{oil} 10-21 ml.h^{-1}). Therefore the ratio between the droplet diameter and the interval to the next droplet is approximately 100:1. This would result in observed sorting frequencies approximately 100-times smaller than that calculated from the theoretical maximum since the droplet travels through a distance of $d_p \sim 2R$ as it undergoes the sorting operation.

As f_{sort} depends on the electric field inhomogeneity $\nabla|\vec{E}|^2$, we derived the analytical form of for both the same-side and cross-side electrode configurations (Supplementary Fig. S6).

The electric field distribution in the case of electrode with the same-side configuration can be modeled as an electric dipole field, considering the tips of the electrodes as point electric charges. Standard textbook derivation[5] dictates that the electric field distribution of a dipole can be written as:

$$\vec{E} = \frac{3gv}{r^2}\sin\theta\cos\theta\hat{i} + \frac{gv}{r^2}(3\cos^2\theta - 1)\hat{j} \tag{10}$$

where r, g, and θ are as defined in FIGGGGGGUU. Using algebra, the magnitude of the electric field $|\vec{E}|$ can be written as:

$$\vec{E} = \sqrt{E_x^2 + E_y^2} = \frac{gv}{r^2}(3\cos^2\theta + 1)^{1/2} \tag{11}$$

Hence, the term $\nabla|\vec{E}|^2$ can be derived as follows:

$$\nabla|\vec{E}|^2 = \frac{-4g^2V^2(1+3\cos\theta^2)}{r^5}\hat{r} + \frac{-6g^2V^2\cos\theta\sin\theta)}{r^4}\hat{\theta} \tag{12}$$

if expressed in polar coordinate form. Hence, the magnitude of $\nabla|\vec{E}|^2$ can be expressed as

$$|\nabla|\vec{E}|^2| = \sqrt{\frac{16g^4V^4(1+3\cos\theta^2)^2}{r^{10}} + \frac{36g^4V^4\cos\theta^2\sin\theta^2}{r^8}} \tag{13}$$

which can be simplified to give:

$$|\nabla|\vec{E}|^2| = 2\frac{g^2V^2}{r^5}\sqrt{4 + 36\cos\theta^4 + 3\cos^2\theta(8+3r^2\sin^2\theta)} \tag{14}$$

The value of $\cos\theta < 1$, hence, we can safely neglect the contribution from the term $\cos^4\theta \ll 1$. The equation then can be reduced to:

$$|\nabla|\vec{E}|^2| = 2\frac{g^2V^2}{r^5}\sqrt{4 + 3\cos^2\theta(8+3r^2\sin^2\theta)} \tag{15}$$

Supplementary Fig. S6 shows that the magnitude $|\nabla|\vec{E}|^2|$ depends on position of the droplet in the sorting chamber: as the droplet traverses the sorting chamber, the magnitude of $|\nabla|\vec{E}|^2|$ varies. The inhomogeneity is extremely large in the region

very close to the proximity of the tip of the electrodes. However, $|\nabla|\vec{E}|^2|$ tapers off rapidly in the region slightly further away from the tip. Indeed, equation (15) shows that $|\nabla|\vec{E}|^2|$ decays with the inverse power of five of the distance to the tip of the electrodes. Supplementary Fig. S6 also reveals that the magnitude of $|\nabla|\vec{E}|^2|$ is always minimum at the y-axis (0,y) and cosθ=0. When cosθ=0, equation (15), reduces to:

$$|\nabla|\vec{E}|^2|_{min} = 4\frac{g^2V^2}{r^5} \tag{16}$$

Similarly, Supplementary Fig. S6 also shows that the magnitude of $|\nabla|\vec{E}|^2|$ is always maximum on the line y=x and y=-x. When the terms $\cos\theta = \frac{1}{2}\sqrt{2}$ and $\sin\theta = \frac{1}{2}\sqrt{2}$, $|\nabla|\vec{E}|^2|$ are maximum equation (15) reduces to:

$$|\nabla|\vec{E}|^2|_{max} = 2\frac{g^2V^2}{r^5}\sqrt{16+\frac{9r^2}{4}} \tag{17}$$

Hence, on average, the magnitude of field inhomogeneity that the droplet experiences is:

$$|\nabla|\vec{E}|^2|_{average} = \frac{g^2(4+\sqrt{64+9r^2})V^2}{2r^5} \tag{18}$$

Since the droplet travels across the sorting chamber, it travels through a distance of $r \sim R$ as it undergoes the sorting operation. Hence, through substitution, we can get:

$$|\nabla|\vec{E}|^2|_{average} = \frac{g^2(4+\sqrt{64+9R^2})V^2}{2R^5} \tag{19}$$

Substituting Equation (19) in place of $|\nabla|\vec{E}|^2|$ in equation (9) gives:

$$f_{sort} = \frac{1}{2R}\frac{2R^2\varepsilon_m Re[f_{CM}]}{3\eta}\frac{g^2(4+\sqrt{64+9R^2})V^2}{2R^5} \tag{20}$$

Which can be rearranged to give:

$$f_{sort} = \frac{\varepsilon_m Re[f_{CM}]}{3\eta}\frac{g^2(4+\sqrt{64+9R^2})V^2}{2R^4} \tag{21}$$

Equation (21) shows the relationship between the sorting frequency, f_{sort}, the distance between the positive and negative electrode, g, the droplet radius, R, and the applied voltage, V. It can be seen that as the applied voltage is increased, the sorting frequency is also increased in quadratic fashion.

However, there is a limit to the voltage that one can apply before the droplet splits owing to Maxwell stress exerted upon the droplet as a result from the applied voltage surmounting the resistance to deformation due to interfacial tension. The magnitude of the electric field, , above which droplets split[6] is given by the expression:

$$|\vec{E}|_{max} = \sqrt{\frac{\gamma}{\varepsilon_0\varepsilon_c R}} \tag{22}$$

where γ, is the interfacial tension, ε_0 is the vacuum permittivity and ε_c is the permittivity of the carrier fluid. Substituting the expression for $|\vec{E}| = -\frac{dV}{dR}$; we can hence write:

$$\frac{-dV_{max}}{dR} = \sqrt{\frac{\gamma}{\varepsilon_0\varepsilon_c R}} \tag{23}$$

$$dV_{max} = -\sqrt{\frac{\gamma}{\varepsilon_0\varepsilon_c R}}dR \tag{24}$$

$$\int dV_{max} = -\int \sqrt{\frac{\gamma}{\varepsilon_0 \varepsilon_c R}} dR \tag{25}$$

$$V_{max} = 2R\sqrt{\frac{\gamma}{\varepsilon_0 \varepsilon_c R}} \tag{26}$$

Hence,

$$V_{max}^2 = \frac{4R\gamma}{\varepsilon_0 \varepsilon_c} \tag{27}$$

Substituting equation (27) into equation (21), then we can obtain the theroretical maximum sorting frequency limited by electrosplitting, f_e:

$$f_e = \frac{\varepsilon_m Re[f_{CM}]}{3\eta} \frac{g^2(4+\sqrt{64+9R^2})V^2}{2R^4} \frac{4R\gamma}{\varepsilon_0 \varepsilon_c} \tag{28}$$

The cross-side configurations can be modelled as a point charge and line charge (Supplementary Fig. S6). The total potential from both point and line charge can be expressed as:

$$V_{total} = \frac{kQ}{\sqrt{x^2+y^2}} - \frac{kQ}{L} \log[\frac{L^2+\sqrt{(w-y)^2+L^2}}{w-y}] \tag{29}$$

Where k ($8.9\ 10^9$ m.F^{-1}) is the Coulomb constant, Q is the electrostatic charge associated with the electrode[5] and L is the length of the electrode 3, as shown in Supplementary Fig. S6. From which, we can then calculate the scalar field of the magnitude of electric field, as follows:

$$|\vec{E}| = \sqrt{\frac{k^2Q^2x^2}{(x^2+y^2)^3} + (\frac{kLQ(1+\sqrt{L^2+(w-y)^2})}{(L^2+\sqrt{L^2+(w-y)^2}\sqrt{L^2+(w-y)^2}(w-y)} + \frac{kQy}{(x^2+y^2)^{3/2}})^2} \tag{30}$$

From the above equation, we can hence derive the $|\nabla|\vec{E}|^2|$ term which determines the DEP force, and thus the sorting frequency.

$$\begin{aligned}
|\nabla|\vec{E}|^2| = \sqrt{}((&-\frac{6k^2Q^2x^3}{(x^2+y^2)^4} + \frac{2k^2Q^2x}{(x^2+y^2)^3} + \frac{6kQxy(-\frac{\frac{kQ(-\frac{1}{\sqrt{L^2+(w-y)^2}}+\frac{L^2+\sqrt{L^2+(w-y)^2}}{(w-y)^2})(w-y)}{L(L^2+\sqrt{L^2+(w-y)^2})}}{} - \frac{kQy}{(w^2+y^2)^{3/2})}}{(x^2+y^2)^{5/2}} - \frac{6k^2Q^2x^2y}{(x^2+y^2)^4})^2 \\
&+2(\frac{kQ(-\frac{1}{\sqrt{L^2+(w-y)^2}}+\frac{L^2+\sqrt{L^2+(w-y)^2}}{(w-y)^2})}{L(L^2+\sqrt{L^2+(w-y)^2})} - \frac{kQ(\frac{2(L^2+\sqrt{L^2+(w-y)^2})}{(w-y)^3} - \frac{1}{\sqrt{L^2+(w-y)^2}(w-y)} - \frac{w-y}{(L^2+(w-y)^2)^{3/2}}(w-y)}{L(L^2+\sqrt{L^2+(w-y)2})} \\
&-\frac{kQ(-\frac{1}{\sqrt{L^2+(w-y)^2}}+\frac{L^2+\sqrt{L^2+(w-y)^2}}{(w-y)^2})(w-y)^2}{L(L^2+\sqrt{L^2+(w-y)^2})^2\sqrt{L^2+(w-y)^2}} + \frac{3kQy^2}{(x^2+y^2)^{5/2}} - \frac{kQ}{(x^2+y^2)^{3/2}}) \\
&(-\frac{kQ(-\frac{1}{\sqrt{L^2+(w-y)^2}}+\frac{L^2+\sqrt{L^2+(w-y)^2}}{(w-y)^2})(w-y)}{L(L^2+\sqrt{L^2+(w-y)^2})} - \frac{kQy}{(x^2+y^2)^{3/2}}))^2)
\end{aligned} \tag{31}$$

Supplementary Fig. S6 shows the distribution of $|\nabla|\vec{E}|^2|$ in the sorting chamber, as calculated using Equation (31). In contrast to the $|\nabla|\vec{E}|^2|$ distribution in the same-side configuration, the $|\nabla|\vec{E}|^2|$ diminishes more slowly in the case of the

cross-side configuration as also observed in the finite-element analysis simulations (Supplementary Fig. S3). Within the sorting chamber, the maximum of $|\nabla|\vec{E}|^2|$ is located at the y-axis (0,y). Hence, if we take limit x approaching to zero, the equation can be simplified to:

$$|\nabla|\vec{E}|^2| = 2\sqrt{}((k^2Q^2(\frac{kLQ(1+\sqrt{L^2+(w-y)^2})}{(L^2+\sqrt{L^2+(w-y)^2})^2(L^2+(w-y)^2)} - \frac{kLQ(1+\sqrt{L^2+(w-y)^2})}{(L^2+\sqrt{L^2+(w-y)^2})\sqrt{L^2+(w-y)^2}(w-y)^2} + \frac{kLQ(2L^2(1+\sqrt{L^2+(w-y)^2})+(3+2\sqrt{L^2+(w-y)^2})(w-y)^2)}{(L^2+\sqrt{L^2+(w-y)^2})(L^2+(w-y)^2)^{3/2}(w-y)^2} - \frac{2kQ\sqrt{y^2}}{y^4})^2 (\frac{L(1+\sqrt{L^2+(w-y)^2})}{(L^2+\sqrt{L^2+(w-y)^2})\sqrt{L^2+(w-y)^2}(w-y)} + \frac{\sqrt{y^2}}{y^3})^2) \quad (32)$$

The width of the sorting chamber is approximately three times the diameter of the droplet, while the droplet itself usually is positioned at $2R$ away from the tip of the pointed electrode. Hence, substituting w = $6R$, and y = $2R$ in the above equation, we can write:

$$|\nabla|\vec{E}|^2| = \frac{1}{2}\sqrt{}(k^4Q^4(\frac{\sqrt{R^2}}{4R^3} + \frac{L(1+\sqrt{L^2+R^2})}{R(R^2+L^2(1+\sqrt{L^2+R^2}))})^2(\frac{\sqrt{R^2}}{R^4} - \frac{4L(1+\sqrt{L^2+R^2})}{(L^2+R^2)(L^2+\sqrt{L^2+R^2})^2} + \frac{4L(1+\sqrt{L^2+R^2})}{R^2(R^2+L^2(1+\sqrt{L^2+R^2}))} - \frac{4L(2L^2(1+\sqrt{L^2+R^2})+R^2(3+2\sqrt{L^2+R^2}))}{R^2(L^2+R^2)^{3/2}(L^2+\sqrt{L^2+R^2})})^2) \quad (33)$$

Rearranging equation (29), we can rewrite:

$$Q = \frac{LV_{total}\sqrt{x^2+y^2}}{k(L-\sqrt{x^2+y^2}\log[\frac{L^2+\sqrt{L^2+(w-y)^2}}{w-y}])} \quad (34)$$

Substituting (34) into (33), and simplifying, we get equation (35):

$$|\nabla|\vec{E}|^2| = \frac{V_{total}^2}{2} \times \sqrt{}(\frac{1}{(L-\sqrt{x^2+y^2}\log[\frac{L^2+\sqrt{L^2+(w-y)^2}}{w-y}])^4)}(L^4(\frac{\sqrt{R^2}}{4R^3} + \frac{L(1+\sqrt{L^2+R^2})}{R(R^2+L^2(1+\sqrt{L^2+R^2}))})^2(\frac{\sqrt{R^2}}{R^4} - \frac{4L(1+\sqrt{L^2+R^2})}{(L^2+R^2)(L^2+\sqrt{L^2+R^2})^2} + \frac{4L(1+\sqrt{L^2+R^2})}{R^2(R^2+L^2(1+\sqrt{L^2+R^2}))} - \frac{4L(2L^2(1+\sqrt{L^2+R^2})+R^2(3+2\sqrt{L^2+R^2}))}{R^2(L^2+R^2)^{3/2}(L^2+\sqrt{L^2+R^2})})^2(x^2+y^2)^2)) \quad (35)$$

Hence, substituting the above equation into Equation (9), we obtain:

$$f_{sort} = \frac{1}{2R}\frac{2R^2\varepsilon_m Re[f_{CM}]}{3\eta}\frac{V_{total}^2}{2} \times \sqrt{}(\frac{1}{(L-\sqrt{x^2+y^2}\log[\frac{L^2+\sqrt{L^2+(w-y)^2}}{w-y}])^4)}(L^4(\frac{\sqrt{R^2}}{4R^3} + \frac{L(1+\sqrt{L^2+R^2})}{R(R^2+L^2(1+\sqrt{L^2+R^2}))})^2(\frac{\sqrt{R^2}}{R^4} - \frac{4L(1+\sqrt{L^2+R^2})}{(L^2+R^2)(L^2+\sqrt{L^2+R^2})^2} + \frac{4L(1+\sqrt{L^2+R^2})}{R^2(R^2+L^2(1+\sqrt{L^2+R^2}))} - \frac{4L(2L^2(1+\sqrt{L^2+R^2})+R^2(3+2\sqrt{L^2+R^2}))}{R^2(L^2+R^2)^{3/2}(L^2+\sqrt{L^2+R^2})})^2(x^2+y^2)^2)) \quad (36)$$

Similar to the case of same-side configuration, the theoretical frequency calculation above dictates the hypothetical sorting frequency limit at a certain applied voltage for cross-side electrode configuration. However, the voltage that can be applied is also limited by the surface tension of the droplet. Hence, if we replace the voltage in the equation above with that of maximum

voltage allowed by surface tension, described by equation (27), we can write the following equation (37), which describes the theoretical maximum sorting frequency limited by electrosplitting, f_e:

$$f_e = \frac{1}{2R}\frac{2R^2\varepsilon_m Re[f_{CM}]}{3\eta}\frac{1}{2}(\frac{4R\gamma}{\varepsilon_0\varepsilon c})\times$$

$$\sqrt{}(\frac{1}{(L-\sqrt{x^2+y^2}\log[\frac{L^2+\sqrt{L^2+(w-y)^2}}{w-y}])^4)}(L^4(\frac{\sqrt{R^2}}{4R^3}+\frac{L(1+\sqrt{L^2+R^2})}{R(R^2+L^2(1+\sqrt{L^2+R^2}))})^2(\frac{\sqrt{R^2}}{R^4}-\frac{4L(1+\sqrt{L^2+R^2})}{(L^2+R^2)(L^2+\sqrt{L^2+R^2})^2}$$

$$+\frac{4L(1+\sqrt{L^2+R^2})}{R^2(R^2+L^2(1+\sqrt{L^2+R^2}))}-\frac{4L(2L^2(1+\sqrt{L^2+R^2})+R^2(3+2\sqrt{L^2+R^2}))}{R^2(L^2+R^2)^{3/2}(L^2+\sqrt{L^2+R^2})})^2(x^2+y^2)^2)) \quad (37)$$

The maximum theoretical sorting frequency, f_e, for same-side and cross-side electrode configurations can be calculated by substituting experimental values into equation (28) and equation (37). The $Re[f_{CM}]$, the Claussius-Mossoti factor, captures the contribution to the DEP due to dielectric constant difference between that of the object and that of the medium. Owing to the fact that the dielectric constant changes as a function of the frequency of the applied electric field, the Claussius-Mossoti factor also changes as a function of the frequency of the applied sorting voltage. However, Ahn et al. reported that the real part of the Claussius-Mossoti factor $Re[f_{CM}]$ is $\sim$1 for frequencies up to $\sim$1 MHz.[1] This frequency is much higher than the voltage frequency used here: 1.4 kV_{pp}, 30 kHz; hence, the $Re[f_{CM}]$ was assumed to be 1. Using a droplet volume of 10 nl, oil viscosity of 18 mPa.s^{-1},[7] relative permittivity of oil of 2,[8] interfacial tension of 10.60 mN.m^{-1},[7] and taking into consideration the spacing of droplets with carrier oil (the ratio between the droplet diameter and the interval to the next droplet is approximately 100:1), the maximum theoretical sorting frequency, f_e, for same-side configuration is 3 droplet.s^{-1}; while that found for cross-side configuration is 46 droplet.s^{-1}. This calculated value for maximum sorting frequency, limited by Maxwell stress, is close to that observed experimentally. Furthermore, plotting f_e for different droplet volumes reveals that with both electrode configurations f_e is inversely proportional to the droplet volume. However, if the droplet volume is >158 pl ($\sim$67 μm diameter), the sorter with the cross-side electrode configuration allows the highest sorting rate, whereas with droplets of <158 pl the sorter with the same-side electrode configuration allows the highest sorting rate.

Skewness analysis of activities distributions from the microplate screening

For each distribution, we measured the skewness of the distribution as the third moment of the distribution (Pearson's moment coefficient of skewness). We obtained a value of -0.5 for the wild-type (O58) distribution (144 replicates) and 1.05 for the sorted fungi distribution (1,262 strains). For a Gaussian distribution, we performed numerical simulation over 10,000 random choices of 144 or 1,262 values and each time measured the skewness. The random selection of 144 values gives a Gaussian distribution of skewness centered around 0 with a standard deviation ($\sigma_{skewness}$) of 0.2. The random selection of 1,262 values leads to a Gaussian skewness distribution centered around 0 with a $\sigma_{skewness}$ of 0.07. These skewness distributions were then used to calculate p-values. The O58 skewness was not significantly different from a normal distribution (p = 0.0124; α = 0.01). However, the sorted fungi distribution has a significant positive skew ($p < 0.00001$; α = 0.01).

References

1. Ahn, K. *et al.* Dielectrophoretic manipulation of drops for high-speed microfluidic sorting devices. *Appl. Phys. Lett.* **88**, 1–3 (2006).

2. Agresti, J. J. *et al.* Ultrahigh-throughput screening in drop-based microfluidics for directed evolution. *Proc. Natl. Acad. Sci. USA* **107**, 4004–4009 (2010).

3. Baret, J.-C. *et al.* Fluorescence-activated droplet sorting (FADS): efficient microfluidic cell sorting based on enzymatic activity. *Lab Chip* **9**, 1850–1858 (2009).

4. Schindler, M. & Ajdari, A. Droplet Traffic in Microfluidic Networks: A Simple Model for Understanding and Designing. *Phys. Rev. Lett.* **100**, 44501 (2008).

5. Gordon, J. & McGrew, R. *Physics for scientists ond engineers 5th edn* (Saunders College Publishing, 2000).

6. Sherwood, J. D. Breakup of fluid droplets in electric and magnetic fields. *J. Fluid Mech.* **188**, 133–146 (1988).

7. Tice, J. D., Lyon, A. D. & Ismagilov, R. F. Effects of viscosity on droplet formation and mixing in microfluidic channels. *Anal. Chim. Acta* **507**, 73–77 (2004).

8. Fuoss, R. M. Dielectric Constants of Some Fluorine Compounds. *J. Am. Chem. Soc.* **60**, 1633–1637 (1938).
